# Evaluating Medicinal Plants for Anticancer Activity

**DOI:** 10.1155/2014/721402

**Published:** 2014-11-13

**Authors:** Elisha Solowey, Michal Lichtenstein, Sarah Sallon, Helena Paavilainen, Elaine Solowey, Haya Lorberboum-Galski

**Affiliations:** ^1^Department of Biochemistry and Molecular Biology, Institute for Medical Research Israel-Canada (IMRIC), Hebrew University Hadassah Medical School, 91120 Jerusalem, Israel; ^2^Louis L. Borick Natural Medicine Research Center, Hadassah Medical Organization, 91120 Jerusalem, Israel; ^3^Arava Institute of Environmental Studies, Kibbutz Ketura, 88840 DN Eilot, Israel

## Abstract

Plants have been used for medical purposes since the beginning of human history and are the basis of modern medicine. Most chemotherapeutic drugs for cancer treatment are molecules identified and isolated from plants or their synthetic derivatives. Our hypothesis was that whole plant extracts selected according to ethnobotanical sources of historical use might contain multiple molecules with antitumor activities that could be very effective in killing human cancer cells. This study examined the effects of three whole plant extracts (ethanol extraction) on human tumor cells. The extracts were from *Urtica membranacea* (Urticaceae), *Artemesia monosperma* (Asteraceae), and *Origanum dayi post* (Labiatae). All three plant extracts exhibited dose- and time-dependent killing capabilities in various human derived tumor cell lines and primary cultures established from patients' biopsies. The killing activity was specific toward tumor cells, as the plant extracts had no effect on primary cultures of healthy human cells. Cell death caused by the whole plant extracts is via apoptosis. Plant extract 5 (*Urtica membranacea*) showed particularly strong anticancer capabilities since it inhibited actual tumor progression in a breast adenocarcinoma mouse model. Our results suggest that whole plant extracts are promising anticancer reagents.

## 1. Introduction

The International Agency for Research on Cancer estimates of the incidence of mortality and prevalence from major types of cancer, at national level, for 184 countries of the world revealed that there were 14.1 million new cancer cases, 8.2 million cancer deaths, and 32.6 million people living with cancer (within 5 years of diagnosis) in 2012 worldwide [1]. By 2030, it is projected that there will be 26 million new cancer cases and 17 million cancer deaths per year [2].

Today, despite considerable efforts, cancer still remains an aggressive killer worldwide. Moreover, during the last decade, novel synthetic chemotherapeutic agents currently in use clinically have not succeeded in fulfilling expectations despite the considerable cost of their development.

Therefore there is a constant demand to develop new, effective, and affordable anticancer drugs [3]. From the dawn of ancient medicine, chemical compounds derived from plants have been used to treat human diseases. Natural products have received increasing attention over the past 30 years for their potential as novel cancer preventive and therapeutic agents [4, 5]. In parallel, there is increasing evidence for the potential of plant-derived compounds as inhibitors of various stages of tumorigenesis and associated inflammatory processes, underlining the importance of these products in cancer prevention and therapy.

Approximately 60% of drugs currently used for cancer treatment have been isolated from natural products [6] and the plant kingdom has been the most significant source. These include* vinca alkaloids, Taxus diterpenes, Camptotheca alkaloids*, and* Podophyllum lignans*. Currently, of 16 new plant-derived compounds being tested in clinical trials, 13 are in phase I or II and three are in phase III [7]. Among these compounds,* flavopiridol*, isolated from the Indian tree* Dysoxylum binectariferum*, and* meisoindigo*, isolated from the Chinese plant* Indigofera tinctoria*, have been shown to exhibit anticancer effects with lesser toxicity than conventional drugs [7]. Medicinal plants constitute a common alternative for cancer treatment in many countries around the world [8, 9]. At this time, more than 3000 plants worldwide have been reported to have anticancer properties. Globally, the incidence of plant-derived products for cancer treatment is from 10% to 40% with this rate reaching 50% in Asiatic patients [9–11]. In Europe alone expenditure for anticancer herbal products is estimated to be 5 billion dollars per year [9].

Israel, with its diverse climatic and geographic conditions, is home to some 2400 plant species [12]. Situated in a transition zone between Mediterranean woodlands in the north, shrubby formations and herbaceous vegetation in the east and south, shrub-steppes and extreme desert areas in the southern Negev, and tropical vegetation in the hottest parts of the country, Israel possesses a great diversity of species many of which are endemic only to this area. With a long history of traditional use spanning many centuries, the medicinal plants of Israel present a unique opportunity for focused screening based on their ethnobotanical use.

In the current study we initially examined the effects of 17 whole plant extracts (ethanol extraction) of Israeli plants on human tumor cell lines as well as human primary cancer cultures. The three most effective plant extracts were then selected for additional research focusing also on the nature of cell death caused by these plant extracts. Our hypothesis was that whole cell extracts might contain multiple molecules with antitumor activities and be very effective in killing human cancer cells.

The 3 plant extracts investigated were* Urtica membranacea* (Urticaceae) (referred to as extract number 5 in the study),* Artemesia monosperma* (Asteraceae) (referred to as extract number 10), and* Origanum dayi Post* (Labiatae) (referred to as extract number 11).

All plants were investigated as part of the* Middle Eastern Medicinal Plant Project (MEMP)*, an initiative of The Natural Medicine Research Center (NMRC) dedicated to the ethnobotanical investigation, domestication, conservation, and reintroduction and focused screening of Israeli medicinal flora [13].

All three selected plant extracts exhibited dose- and time-dependent killing capabilities in various human derived hematological and solid tumor cell lines and in primary cultures established from patients' biopsies. The killing activity was specific toward tumor cells, as the extracts had no effect on primary cultures of healthy human cells (lymphocytes and fibroblasts). Several experiments were carried out to characterize the plant extracts' activities. Using various methods it was found that, using whole plant extracts, cell death was caused via apoptosis.

Plant extract 5 also demonstrated strong anticancer capabilities by inhibiting actual tumor progression in a breast adenocarcinoma mouse model. Our results suggest that whole plant extracts are promising anticancer reagents.

## 2. Materials and Methods

### 2.1. Plant Selection

Plants were selected for screening using a focused method based on ethnobotanical information derived from The Natural Medicine Research Center's (NMRC) database of medicinal plantscontaining the traditional and folk medicine uses of over 500 Israeli species and from data mining techniques using additional sources including medieval pharmacopeias and medical encyclopedias translated from original Latin, Hebrew, and Arabic texts.

Selected plants were collected from wild sources or harvested from domesticated plants derived from seeds obtained from wild plants. Domestication was carried out at the MEMP cultivation site in Kibbutz Ketura, in Israel's southern Arava region (arid and desert species) and at Moshav Noam on the coastal plain (Mediterranean species). Plants were cultivated without the use of fertilizers or pesticides using shaded net houses and drip irrigation. Collection of wild plants was carried out in conjunction with Ms. Hagar Leschner and Dr. Ori Fragman-Sapir. All plant material was definitively identified by either Dr. Ori Fragman-Sapir or Ms. Hagar Leschner and voucher herbarium specimens deposited at the Jerusalem Botanical Gardens.

### 2.2. Plant Extracts (Ethanol Extraction)

Freshly harvested plants were air-dried at room temperature and extracted with ethanol (50% v/v, 10 v per gram weight) by vigorous stirring in a covered beaker for 24 h at room temperature after which the process was repeated. The supernatant of both extracts was filtered and ethanol evaporated in a chemical hood for 4 days with the evaporated extract frozen at −70°C followed by lyophilization till dryness. The dried extract was kept at 4°C. Stock solutions of the plant extracts (200 mg/mL) were prepared by weighing the powder and dissolving it in 10% DMSO/PBS. The solution was divided to aliquots and kept at −20°C, until used.

### 2.3. Cell Lines

The human cancer cell lines HepG2, OVCAR 3, 293, A549, T24P, and SU-DHL-1 and mouse cell lines D122 and B16 were grown in DMEM supplemented with 10% fetal calf serum (FCS), 1 mM L-glutamine, 100 units/mL penicillin, and 100 *μ*g/mL streptomycin.

The human cancer cell lines Hec1A, Karpas, HUT102, Colo205, LNCaP, MCF-7, YC, and OSTRA were grown in RPMI 1640 supplemented with 10% FCS and antibiotics as above. E0771 mouse cells were grown in F-12 (HAM'S) medium containing 10% FCS, 1 mM L-glutamine, 10 mM HEPES, and antibiotics as above.

Primary human cancer cultures were grown in RPMI 1640 medium containing 1% nonessential amino acids, 1% sodium pyruvate, 1% sodium bicarbonate, 20% FCS, 10 *μ*M *β*-mercaptoethanol, 1 mM L-glutamine, and antibiotics as above.

All media and supplements were purchased from Biological Industries (Bet Ha'emek, Israel). The cells were kept in a humidified atmosphere with 5% CO_2_ at 37°C. All cultures were tested for mycoplasma contamination and were found to be negative.

### 2.4. Isolation of Human Primary Lymphocytes from Healthy Donors

Human peripheral blood mononuclear cells (PBMCs) from healthy donors (obtained from the Blood Bank-Hadassah Medical Center) were isolated on a Ficoll-Isopaque gradient (density 1.077; Sigma-Aldrich, St. Louis, MO, USA). The isolated PBMCs were then counted and checked for viability using Trypan blue and used on an individual basis. To activate the lymphocytes, all mononuclear cells were grown in RPMI 1640 supplemented with 10% (v/v) FCS, 2 mM L-glutamine, 100 units/mL penicillin, 100 *μ*g/mL streptomycin, 10 *μ*M *β*-mercaptoethanol, and phytohemagglutinin (PHA 20 *μ*g/mL) for 3 days. The activated cells were washed and resuspended in the presence of 10 units/mL of recombinant IL-2 (PeproTech EC Ltd., London, UK) to maintain cell viability. Naïve lymphocytes were grown in RPMI 1640 supplemented with 10% (v/v) FCS, 2 mM L-glutamine, 100 units/mL penicillin, 100 *μ*g/mL streptomycin, and 10 *μ*M *β*-mercaptoethanol.

### 2.5. Primary Human Cancer Cultures

Fresh tissue specimens were taken from cancer patients undergoing therapeutic debulking procedures. All tissue specimens were washed several times with Leibovitz (L15) medium, minced, and subjected to enzymatic proteolysis for 2 h at 37°C with gentle shaking in Leibovitz medium containing collagenase type I (200 units/mL), hyaluronidase (100 units/mL) (Sigma-Aldrich), penicillin (1000 units/mL), streptomycin (1 mg/mL), and amphotericin B (2.5 *μ*g/mL). Tissue preparations were centrifuged for 10 min at 200 g, and the pellets were suspended in RPMI 1640 medium containing all supplements and plated in 100 mm petri dishes. After 1–3 weeks, when the cultures had reached a density of 8 × 10^6^ cells/plate, histopathological diagnoses and cell viability assays (see below) were performed.

### 2.6. Cell Viability

10^4^/100 *μ*L/well cells growing in suspension or 5 × 10^3^/100 *μ*L/well adherent cells were seeded and treated with increasing concentrations of the plant extracts for 48–72 h, after which CellTiter-Blue reagent (Promega, Madison, WI, USA) was added according to the manufacturer's instructions to determine cell survival. All treatments were performed in triplicate.

### 2.7. Cell Cycle Analysis by Propidium Iodide (PI) Staining

0.3 × 10^6^/3.5 mL Hec1A cells were treated with the plant extracts (1.5 mg/mL, final concentration), for 6 to 48 h. Samples were removed for assaying cell viability, and the remaining cells were centrifuged at 500 g for 6 min at 4°C, washed with cold PBS, and resuspended in 0.5 mL of PI hypotonic solution (50 *μ*g/mL PI; 0.1% sodium citrate; 0.1% triton X100). After overnight incubation at 4°C, cell cycle analysis of the cells was performed using FACScan and the CellQuest program (Becton Dickinson).

### 2.8. *In Vitro* Caspase 3 Activity Assay

10^5^/100 *μ*L/well Colo205 cells were treated with the plant extracts (1.5 mg/mL, final concentration), for 24–48 h. Caspase 3 activity within the cells was assessed by Apo-ONE Homogeneous Caspase 3/7 Assay Kit (Promega). Experiments were carried out in parallel with cell viability assays.

### 2.9. Western Blot Analysis

Samples were separated on 12% SDS-PAGE gels. The proteins were then electrotransferred onto Immobilon-P transfer membrane (Millipore, Millipore, Bradford, MA, USA) and blotted with anti-PARP (Cell Signaling Technology, Inc, Boston, USA; 1 : 1,000). Band visualization was achieved using an enhanced chemiluminescence kit (ECL, Biological Industries).

### 2.10. DNA Laddering

0.5 × 10^6^/3 mL Colo205 cells were seeded and the plant extracts were added (1.5 mg/mL, final concentration) for 24, 48, or 72 h. The cells were then collected, washed twice with PBS, resuspended in 4 mL of lysis buffer (15 mM Tris-HCl pH 7.4, 3 mM EDTA pH 8.0, 150 mM NaCl, 0.2% SDS, 10 *μ*g/mL proteinase K, and 50 *μ*g/mL RNAse), and incubated over night at 37°C. DNA was then extracted by the following procedure: 4 mL phenol/chloroform (1 : 1 ratio to lysis buffer volume) was added, and the solution was centrifuged at 3150 g for 5 min at room temperature. The upper phase was isolated and 1 : 1 volume of chloroform was added. The solution was then centrifuged again under the same conditions and the upper phase was collected. NaCl concentration was adjusted to 0.5 M. Two volumes of absolute cold (−20°C) ethanol were added and the solution was incubated at −70°C for 1 h, to allow the DNA precipitate to form. The precipitate was then isolated by centrifugation at 10,000 g for 30 min at 4°C. The pellet was washed twice with 70% cold ethanol, air-dried, and resuspended in 10 mM Tris, 1 mM EDTA, pH 8.0. The final concentration was measured by nanodrop machine. 10 *μ*g DNA of each sample was loaded on a 1.5% agarose gel.

### 2.11. Real-Time PCR Analysis of Apoptosis-Related mRNAs in Colo205 Treated Cells

0.5 × 10^6^/3.5 mL Colo205 cells were treated with 1.5 mg/mL (final concentration) of extracts 5, 10, and 11 for 3–24 h. Total mRNA was extracted and reverse-transcribed into cDNA using Verso cDNA kit (Thermo Specific, Epsom, Surrey, UK). Individual mRNA levels were quantified using Real-Time PCR (Applied Biosystems, Foster City, CA, USA). Each 2 *μ*L sample contained 1 *μ*L primers (10 ng), 10 *μ*L SYBR Green (Applied Biosystems), and 7 *μ*L H_2_O in a total volume of 20 *μ*L per sample. The primers were as follows:

 Bax (exons 4-5, 116 bp) Sense: TCT GAC GGC AAC TTC AA CTG;
 Antisense: CAG CCC ATG ATG GTT CTGA;
 Bcl2 (exons 2-3, 134 bp) Sense: CCC CTG GTG GAC AAC ATC;
 Antisense: CAG CCA GGA GAA ATC AAA CAG;
 Caspase 3 (exons 7-8, 133 bp) Sense: GAA CTG GAC TGT GGC ATT GA;
 Antisense: CCT TTG AAT TTC GCC AAG AA;
 G6PD (exons 6-7, 283 bp) Sense: TCT ACC GCA TCG ACC ACT ACC;
 Antisense: GCG ATG TTG TCC CGG TTC.

The data was analyzed by the primer express program (Applied Biosystems).

### 2.12. Effect of Plant Extract 5 on Breast Cancer Growth in Mice

C57BL/6JolaHsd mice were radiated with 500RAD/5 GREY, left for one day to recover, and then subcutaneously injected with 10^5^ E0771 cells, in the lower back. Treatment started one day after tumor induction and consisted of daily i.p. injections of 150 *μ*g (in 300 *μ*L) extract 5, for 12 days. Control mice were injected with an equivalent volume of DMSO/PBS at the same concentration as in extract 5. At the end of the experiment, mice were sacrificed; tumors were removed and weighed.

## 3. Ethics Statement

Human peripheral blood mononuclear cells from healthy donors and biopsies from cancer patients were obtained according to the guidelines approved by the Hadassah Hospital Ethics Committee (Jerusalem, Israel). All subjects provided written informed consent.

All procedures involving mice were approved by the Hebrew University of Jerusalem Institutional Animal Care and Use Committee, approval number MD-07-10429-5. This study was conducted in accordance with the NIH Guide for the Care and Use of Laboratory Animals (NIH approval number OPRR-A01-5011). The mice were housed in cages with autoclaved bedding and equipped with filter caps under containment protocols with up to five animals per cage. A 12 h light/dark cycle was maintained, and mice had access to water and rodent laboratory chow ad libitum.

## 4. Results

### 4.1. Screening Plants for Their Anticancer Activity

17 plants were initially selected and tested for anticancer activity based on their historical and traditional use in treating cancer. Whole plant extracts (ethanol extracted) were prepared, coded as plant extract numbers 1–17, and tested for their potential as anticancer agents. We tested a variety of cancer cell lines originating from various human tumors including hematopoietic tumors such as T cell lymphoma and leukemia, as well as solid tumors such as colon, renal, breast, bladder, endometrial, and others (Table 1). This was done by closely monitoring the viability of cultured human cells exposed to the plant extracts.

The plant extracts (added at 3 mg/mL, final concentration) inhibited the growth of the various human tumor cell lines eventually leading to cell death. Efficacy of cell death varied, depending on the specific plant extract (Figure S1 A–C in Supplementary Material available online at http://dx.doi.org/10.1155/2014/721402).

Specificity of plant extracts toward cancer cells was also evaluated. Normal human lymphoblast (EBV transformed) cells (OSTRA and YC cells; Table 1) were not affected by the treatment with the plant extracts (Figure S1 A–C). Thus, the plant extracts are cytotoxic to established human cancer cell lines but have no effect on healthy human cells. Based on repeated assays, the three most effective anticancer plant extracts, 5, 10, and 11, were chosen for further studies.

### 4.2. Plant Extract Induced Cell Death Was Time- and Concentration-Dependent

**Figure 1:**
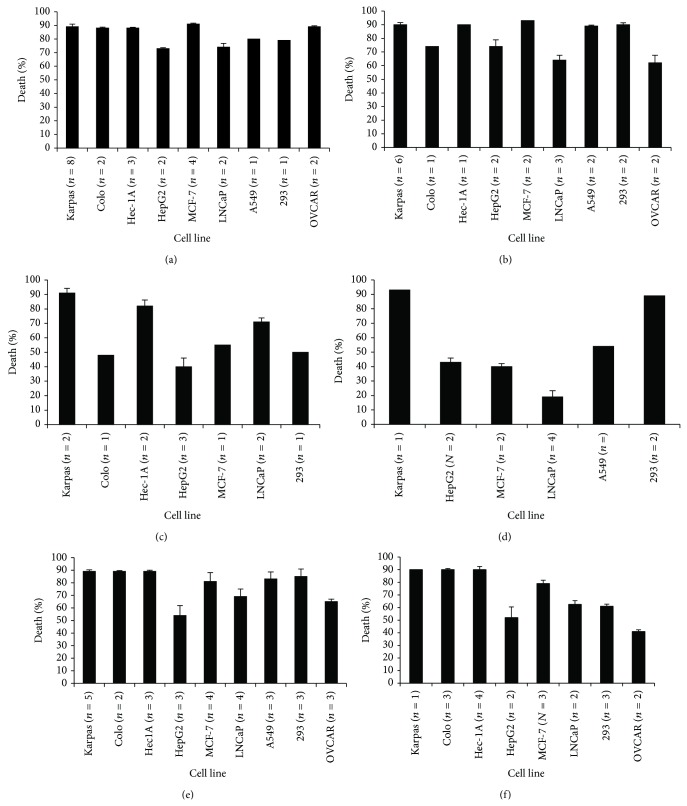
Effect of whole plant extracts 5, 10, and 11 on growth of human tumor cell lines—concentration dependency. Whole plant extracts 5 ((a), (b)), 10 ((c), (d)), and 11 ((e), (f)) were added to the indicated cells (10^5^ cells/100 *μ*L) at 1.5 mg/mL ((a), (c), (e)) or 0.75 mg/mL ((b), (d), (f)) for 48 h, after which cell viability was determined as described in Materials and Methods. Results are expressed as percentage of death caused by each plant extract, as compared to control cells (treated with DMSO/PBS at the same concentration as the plant extracts). *n* = number of independent experiments performed on each tested cell line. Each column represents the mean ± standard deviation of 1 to 8 independent experiments.

**Figure 2:**
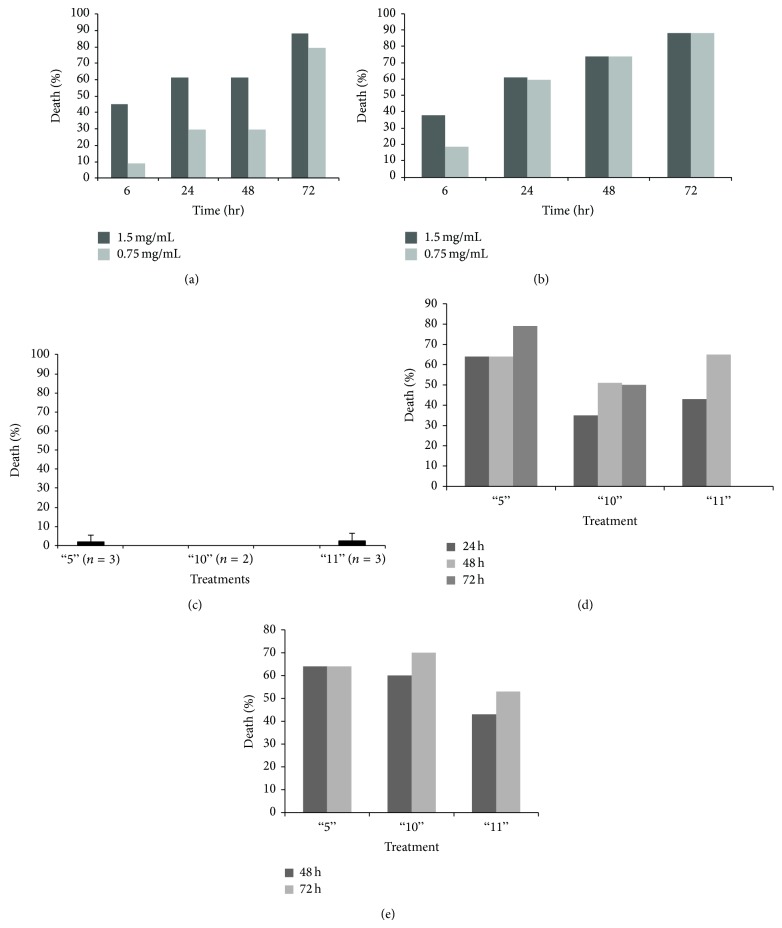
Effect of whole plant extracts 5, 10, and 11 is time-dependent and specific. (a)-(b) Effect of whole plant extracts is time dependent. Plant extracts 5 and 11 were added at two different concentrations (1.5 mg/mL and 0.75 mg/mL) to Hec1A cells for various incubations periods and cell viability assays were performed as described in Materials and Methods. Results are expressed as percentage of death caused by each plant extract as compared to control cells (treated with DMSO/PBS at the same concentration as the plant extracts). Each column represents one representative out of 2–4 independent experiments performed. (c) Effect of whole plant extracts is specific to tumor cells. Whole plant extracts 5, 10, and 11 were added to human primary lymphocytes from healthy donors (obtained from Blood Bank-Hadassah Medical Center and used on an individual basis; 10^5^ cells/100 *μ*L) at 1.5 mg/mL for 72 h, after which cell viability was determined as described in Materials and Methods. Results are expressed as percentage of death caused by each plant extract, as compared to control cells (treated with DMSO/PBS at the same concentration as the plant extracts). The numbers in parentheses (*n*) are the number of individuals whose lymphocytes were tested with the three plant extracts. Each column represents the mean ± standard deviation of 2 to 3 independent experiments. (d)-(e) Primary cultures were established from biopsies of two different cancer patients (colon carcinoma and breast cancer patients) and plant extracts 5, 10, and 11 were added to the cell cultures (10^5^ cells/100 *μ*L; 1.5 mg/mL) for different incubation periods and cell viability assays were performed as described in Materials and Methods.

The 3 most effective anticancer plant extracts 5, 10, and 11, were first tested for their effect on cancer cell lines at two lower concentrations. As can be seen in Figure 1, the three plant extracts inhibited cell growth also at lower concentrations, with different efficacy depending on the specific plant extract and the cancer cell line tested. Inhibition of cell growth was also time-dependent, as demonstrated in Figures 2(a) and 2(b). Plant extracts 5, 10, and 11 were tested also on primary lymphocytes from healthy control donors and showed no killing activity on these cells (Figure 2(c)). Thus, plant extracts 5, 10, and 11 exhibited time- and concentration-dependent anticancer activity while having no effect on primary cell cultures from healthy humans.

### 4.3. Effect of the Plant Extracts on Primary Cultures Obtained from Cancer Patients

To study the cytotoxicity of our plant extracts against cells resembling the original* in vivo* tumors as closely as possible, primary cultures were established from biopsies of two different cancer patients (colon carcinoma and breast cancer patients). As can be seen in Figures 2(d) and 2(e), extract 5 affected cell growth very quickly, as was seen with the established cell lines. Extracts 10 and 11 caused cell death more slowly; however, their killing activity increased 72 h after the start of treatment. It should be noted that although cell growth of the primary cell cultures was inhibited by about 60%, this killing activity is much lower than that measured in established cell lines (see Figure S1 and 1-2). This may be explained by the fact that primary cultures are most probably not homogenous but rather a mixed population of cells, including healthy normal cells for which the plant extracts were not cytotoxic.

### 4.4. Mechanism of Cytotoxicity

#### 4.4.1. Effect of Plant Extracts on Cell Cycle of Hec1A Endometrium Cancer Cells

In late stages of apoptosis, cells split to form apoptotic bodies. Each apoptotic body contains only part of the original cell's DNA content. When stained with PI, this population is known as the sub-G1 population and is characterized by having a DNA content of less than 2n chromosomes. In addition, apoptotic cells demonstrate specific morphological changes such as chromatin condensation and plasma membrane blebbing. These changes cause the cell to be more granular and larger in size when analyzed by FACS.

**Figure 3:**
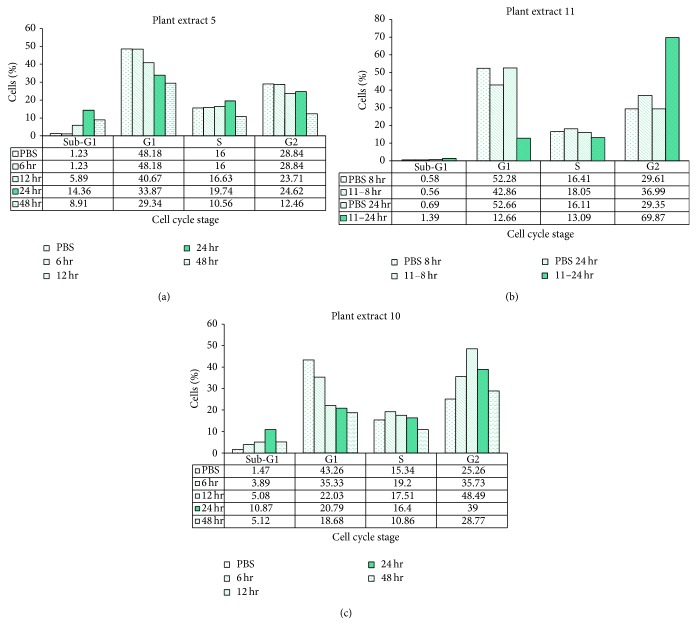
Effect of whole plants extracts 5, 10, and 11 on the cell cycle of Hec1A tumor cells. These graphs illustrate the effect of plant extracts 5, 10, and 11 on the cell cycle, as demonstrated by PI staining. Hec1A cells (3 × 10^5^/3.5 mL) were treated with 1.5 mg/mL plant extract 5, 10, or 11 for various time periods. The cells were stained with 50 *μ*g/mL PI, and the DNA content was analyzed by FACS analysis, as described in the text (see Materials and Methods).

In order to demonstrate the effect of our plant extracts on the cell cycle, endometrial adenocarcinoma HeC1A cells were treated with the three extracts (1.5 mg/mL each, final concentration) for different time periods and analyzed by FACS. Results are shown in Figure 3.

Extract 5 caused ~13% increase in the sub-G1 cell population after 24 h. This increase was accompanied by a 14% decrease in cells at G1 phase (Figure 3(a)). Extract 11 caused a dramatic increase (40%) in cell numbers at G2 with no apparent change in the sub-G1 cell population (Figure 3(b)). Extract 10 caused an increase in the sub-G1 cell population (9.4% increase, Figure 3(c)), a decrease (from 43.26% to 20.79%) in cells at G1 and a considerable increase (from 25.26% to 39%) in cells at G2 (Figure 3(c)). These results indicate that extracts 5 and 10 cause classic apoptotic cell death, while extract 11 is causing death via a different mechanism.

#### 4.4.2. Increase in Intracellular Caspase 3 Activity Following Treatment with the Plant Extracts

**Figure 4:**
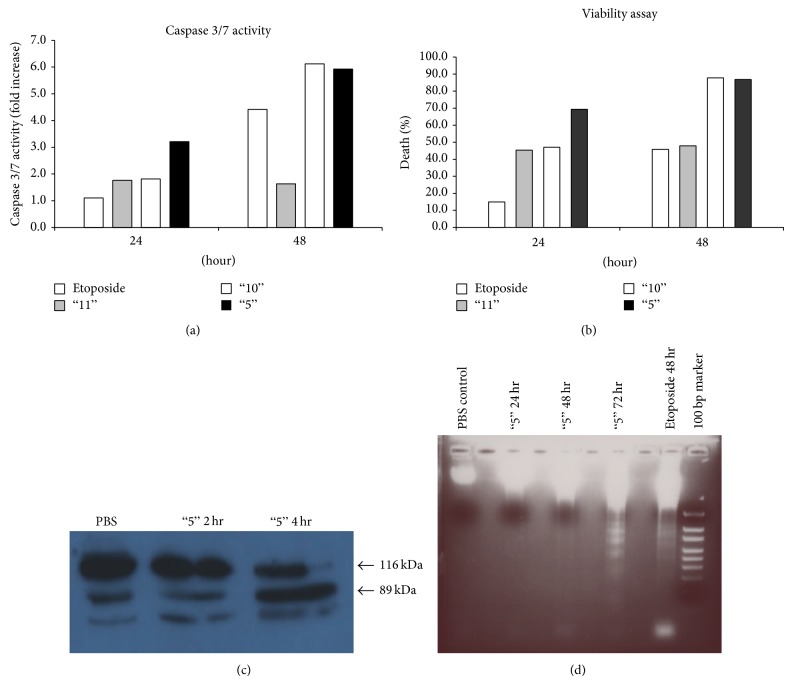
Whole plant extracts induce cell death of tumor cells via apoptosis. (a)-(b) Caspase 3 activity (a) and cell death (b) of Colo205 tumor cells exposed to plant extracts 5, 10, and 11 for 24 or 48 h. Caspase 3 activity was determined as described in Materials and Methods. The columns depict changes in caspase 3 activity as the fold increase for each plant extract at both time periods. (c) Colo205 cells were treated with extract 5 (1.5 mg/mL), for 2 or 4 h, and cell lysates were prepared. After treatment, total protein extracts from the cells were separated by SDS-PAGE and analyzed by western blot analysis using anti-human PARP antibodies. Arrows indicate the full-length PARP protein (116 KDa; upper arrow), and its cleaved product (89 KDa; lower arrow), following exposure of the cells to plant extract 5. (d) DNA fragmentation in Colo205 cells exposed to plant extract 5. For detection of DNA fragmentation, DNA was extracted from Colo205 cells exposed to Etoposide (50 *μ*L/mL; for 48 h) or extract 5 (1.5 mg/mL) for various time periods. From left to right: DNA extracted from control Colo205 cells, not exposed to any inducer (48 h; line 1); DNA extracted from cells exposed to extract 5 for 24 h (line 3), 48 h (line 5), 72 h (line 7), or Etoposide (line 9).

Intracellular caspase 3 activation is a key stage in the apoptotic pathway. Hence, we tested the effect of treatment with our plant extracts on intracellular caspase 3 enzymatic activity. Human colon cancer cells (Colo205) were treated with the plant extracts or with Etoposide, a known inducer of apoptosis, and caspase 3 enzymatic activity within the cells was measured using a synthetic substrate (Figures 4(a) and 4(b)). In these experiments, total caspase 3 activity from the entire cell population was measured. Since caspase 3 activity rises while cells die and cell numbers drop, it was essential to normalize caspase 3 activity to the number of cells, to obtain more accurate results. Therefore, identical samples were analyzed simultaneously for cell viability (Figure 4(b)). The results here are expressed as the fold increase in caspase 3 activity in treated cells compared to cells added with DMSO/PBS (at the same final concentration as in the plant extracts; control cells).

Treatment with extract 5 increased caspase 3 enzymatic activity 3.2-fold after 24 h and 5.9-fold after 48 h (Figure 4(a)), suggesting an induction of apoptosis. A similar increase in caspase 3 activity was observed following treatment with extract 10 (1.8-fold after 24 h and 6.1-fold after 48 h; Figure 4(a)). Plant extract 11 hardly increased caspase 3 activity in the treated cells (Figure 4(a)). In Colo205 cells exposed to Etoposide as a positive control for apoptosis, caspase 3 increased 4.4-fold after 48 h of treatment (Figure 4(a)). The results suggest that plant extracts 5 and 10 caused an apoptotic, caspase-3-dependent death, while extract 11 caused death by a caspase-3-independent mechanism. These results are in accordance with the extracts effects seen on cell cycle (see Figure 3).

#### 4.4.3. Plant Extract 5 Causes PARP Cleavage in Colo205 Cells

Another way of demonstrating that death induced by the plant extracts is via classic caspase-3-dependent apoptosis is to follow one of caspase 3's substrates, the poly(ADP-ribose) polymerase (PARP) protein that undergoes cleavage upon an apoptotic signal. Colo205 cells were treated with extract 5, for 2 or 4 h, and cell lysates were prepared. As can be seen in Figure 4(c), following treatment the PARP protein was cleaved into its known shorter fragment of ~89 kDa. This confirmed that death induced by plant extract 5 is indeed via caspase-3-dependent apoptosis.

#### 4.4.4. Apoptosis Induced by Plant Extract 5 as Demonstrated by DNA Laddering

DNA fragmentation is a well-known biochemical feature of apoptosis. One of the numerous proteins cleaved by caspase 3 is DFF45. Upon cleavage, DFF45 releases and activates the endonuclease DFF40, which breaks DNA strands in the internucleosomal regions, producing fragments of various lengths; these fragments form a unique laddering pattern.

Colo205 cells were treated with plant extract 5 (1.5 mg/mL, final concentration) for various time periods. Etoposide was used as a positive control. Following treatment with extract 5 for 72 h, Colo205 cells exhibit DNA laddering patterns similar to those obtained using Etoposide, indicating that our extract 5 induced apoptotic DNA fragmentation (Figure 4(d)).

#### 4.4.5. The Effect of Plant Extracts on the Expression Levels of Apoptotic Proteins

**Figure 5:**
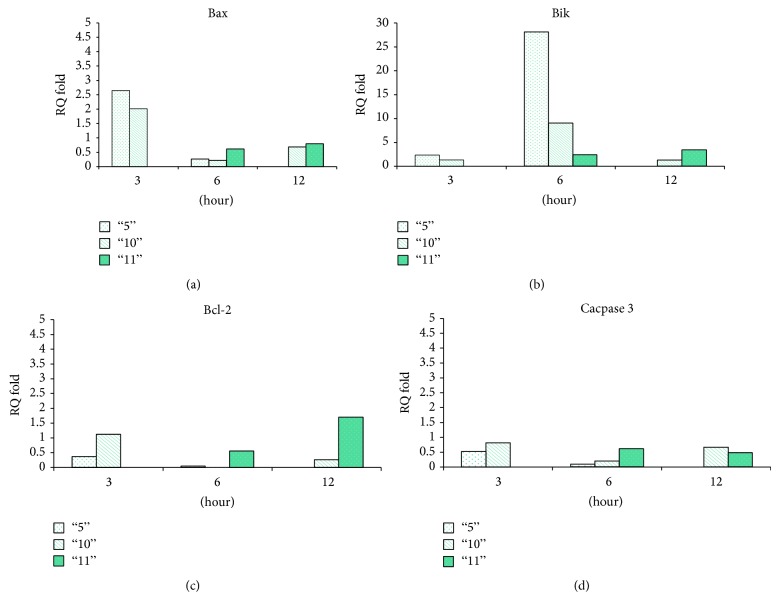
Changes in the expression levels of cellular apoptotic proteins following treatment with whole plant extracts. (a)–(d) Colo205 tumor cells were treated with the three plant extracts (1.5 mg/mL each; final concentration) then analyzed for mRNA levels by PCR for Bax protein (a), Bik protein (b), Bcl2 protein (c), and caspase 3 protein (d). The results are expressed as the fold elevation in mRNA levels compared to control cells (treated with DMSO/PBS at the same final concentration as the plant extracts).

We examined the effect of the plant extracts on the expression levels of apoptotic proteins using real time PCR. As can be seen in Figure 5, treatment with plant extract 5 caused a 2.5-fold increase in cellular mRNA levels of Bax. The increase in cellular expression was dramatic for Bik-mRNA levels (>25-fold) peaking at 6 h. In contrast, expression of Bcl-2 declined following treatment. Plant extract 10 caused similar changes in the expression of the tested apoptotic proteins (2-fold increase in Bax mRNA, 9-fold in Bik mRNA, and a decrease in Bcl-2 mRNA; Figure 5). Extract 11 caused a different response in Colo205 cells, a late (12 h) and mild increase in both Bax and Bik mRNAs (1.5-fold and 1.7-fold, resp.) and a constitutive increase in Bcl-2 mRNA.

### 4.5. Effect of Plant Extract 5 on Breast Cancer Growth in Mice

In order to test the effect of the extracts in an* in vivo* mouse model, we first checked their effect on three mouse cancer cells: B16 melanoma, D122 lung carcinoma, and E0771 breast carcinoma cells. Plant extract 5 was found to be the most effective on E0771 cells (results not shown) and thus was chosen to be tested* in vivo* in the E0771 breast cancer model in mice.

First the nonspecific toxicity of extract 5 was tested on male C57BL/6JolaHsd mice by single i.p. injections of increasing amounts of extract 5. The mice were followed for three weeks for vital signs and weighed twice a week. Injecting up to 150 *μ*g of plant extract 5 was found to be safe with no toxic effects on the mice.

**Figure 6:**
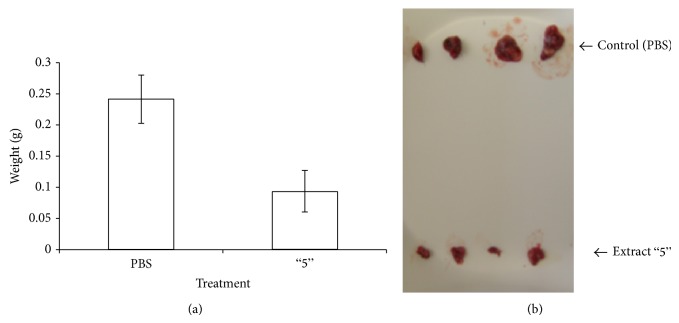
Plant extract 5 inhibits tumor growth* in vivo*, in a mouse breast cancer model. C57BL/6JolaHsd mice were injected s.c. with 10^5^ E0771 cells, in the lower back. Treatment started one day after tumor induction and consisted of daily i.p. injections of 150 *μ*g (in 300 *μ*L) extract 5, for 12 days. Control mice were injected with an equivalent volume of DMSO/PBS at the same final concentration as the injected plant extract 5. At the end of the experiment, mice were sacrificed; tumors were removed and weighed. Each column represents average weight of tumors removed from 10 mice ± SE (a). Images of tumors removed from treated and control mice (b).

Next, C57BL/6JolaHsd mice were injected with E0771 mouse breast cancer cells and treated with extract 5 (see methods, Section 2.12). As demonstrated in Figure 6(a), tumors of mice treated with extract 5 weighed about 50% less than those of mice receiving only DMSO/PBS (vehicle).  Figure 6(b) shows the tumors from both groups, emphasizing the significant difference in tumor size of the treated mice as compared to the control mice. Thus, plant extract 5 inhibits tumor growth* in vivo*, in a mouse breast cancer model.

## 5. Discussion

Medicinal plants have been traditionally used in folk medicine for centuries as natural healing remedies with significant proven therapeutic effects in many areas including prevention of cardiovascular diseases and anti-inflammatory, antimicrobial, and anticancer activity. In addition, the emergence of resistance to cancer chemotherapy has forced researchers to turn to natural products of plant and marine origin.

Although many compounds isolated from plants are being rigorously tested for their anticancer properties, it is becoming increasingly recognized that the beneficial effects of plants are due to a complex interplay of the composite mixture of compounds present in the whole plant (additive/synergistic and/or antagonistic) rather than constituent single agents alone [14, 15]. With this mind-set, we sought to undertake a detailed evaluation of the* in vitro* and* in vivo* anticancer activity of a number of Israeli whole plant extracts.

We investigated the anticancer properties of mainly 3 plant extracts native to Israel:* Urtica membranacea* (Urticaceae; extract number 5),* Artemesia monosperma* (Asteraceae) (extract number 10), and* Origanum dayi Post* (Labiatae) (extract number 11).

*Artemisia monosperma*.* Artemisia monosperma* (plant extract 10) is a glaucous, glabrous, dwarf shrub that grows widely in the sandy vegetation of the western Negev, northern Sinai, and coastal sands of Israel. The genus* Artemisia* with some 400 species mainly in the Northern hemisphere is known for its extensive use in traditional medicine and for its wide range of biological activities including antimalarial, cytotoxic, antihepatotoxic, antibacterial, antifungal, and antioxidant activity [16]. Major classes of phytoconstituents found in the* Artemisia* genus include terpenoids, flavonoids, coumarins, caffeoylquinic acids, sterols, and acetylenes [16].

Analysis of essential oil from the stem and leaf of* A. monosperma* has revealed 130 components [17]. An eudesmane sesquiterpene present in* A. monosperma* [18] has been associated with apoptotic cell death in the human melanoma A375 cell line [19].

Several other compounds isolated from* A. monosperma* have demonstrated specific* in vitro* anticancer activity against colorectal and breast cancer cell lines by polyacetylene dehydrofalcarindiol [20]. Capillin (1-phenyl-2,4-pentadiyne), another polyacetylene found in* A. monosperma*, has been shown to induce apoptosis in several human tumor cell lines including colon HT29, pancreatic MIA PaCa-2, epidermoid carcinoma of the larynx HEp-2, and lung carcinoma A549 cells [21].

In this study we demonstrated that* A. monosperma* whole plant extract has anticancer activity against at least 10 different human tumor cells and two primary tumor cultures, causing these tumor cells to die via apoptosis.

*Origanum dayi Post*.* Origanum dayi Post* (plant extract 11) is a perennial subshrub endemic to the desert regions of Israel including the northern Negev and the Judean desert. Members of the* Origanum* genus, of which there are some 10 Mediterranean and Saharo-Arabian species, are aromatic and have been widely used since antiquity as culinary herbs and for medicinal purposes [13, 22]. One of the main components of the essential oil of* O. dayi* 1,8-cineole has been associated with suppression of growth in human leukemia Molt 4B and HL-60 cell lines due to induction of apoptosis by this compound [23].

Carvacrol, a monoterpene found in smaller quantities in* O. dayi*, has been shown to be a potent inhibitor of cell growth in the human non-small-cell lung cancer (NSCLC) cell line, A549 [24]. Whole plant extracts of* O. dayi* have also been recently associated with an* in vitro* antiproliferative effect in HepG2 cells [25].

In our study,* O. dayi* whole plant extract exhibited strong anticancer activity against human tumor cell lines as well as primary tumor cultures. However, death induced by its extract caused the tumor cells to die, most probably, via a caspase-3-independent mechanism. It should be pointed out that one of the main problems of conventional anticancer therapy is multidrug resistance (MDR), whereby cells acquire resistance to structurally and functionally unrelated drugs following chemotherapeutic treatment. One of the main causes of MDR is overexpression of the P-glycoprotein transporter. In addition to extruding the chemotherapeutic drugs, it also inhibits apoptosis through the inhibition of caspases. Thus,* O. dayi* whole plant extract could overcome MDR and should be tested for its anticancer potential against human tumor cells resistant to chemotherapeutic drugs.

*Urtica membranacea*.* Urtica membranacea* (plant extract 5), one of 4 species of the* Urtica* genus found in Israel, is characterized by large leaves and stinging hairs and is a common annual in Mediterranean woodlands and shrublands.* U. membranacea* has been relatively little investigated compared to other more widely researched members of the genus, which contains some 40 species found mainly in temperate regions with a long history of medicinal use including arthritis, hay fever, tonics, eczema, haemorrhoids, hyperthyroidism, bronchitis, and cancer [26].

Antitumour activity has been shown in a number of studies on* U. dioica *particularly in prostate growths including antiproliferative properties on human prostate cancer cells [27], inhibition of membrane Na^+^, K^+^ ATPase activity in benign prostatic hyperplasia through the steroidal component of* U. dioica* root [28], and modulation of sex hormone-binding globulin to its receptor on human prostatic membranes [29]. Nevertheless, as mentioned, very little research has been performed on* U. membranacea*.

Research on plants used in Calabria (southern Italy) in folk plant medicine was carried out by Passalacqua et al. for twenty years. The use of 104 taxa distributed into 42 families has been described. One of the major findings was that* U. membranacea* is used for tussis and tonsillitis [30]. No effect on cancer was however reported.

Here we show that a whole extract of* U. membranacea* is highly effective in killing a large number of human tumor cells as well as human primary cultures (Figures 1-2). In addition, death is caused via apoptosis (Figures 3–5). Moreover,* U. membranacea* extract inhibited tumor growth* in vivo*, in a mouse breast cancer model (Figure 6).* U. membranacea*, similar to* O. dayi*, is edible and nontoxic to humans.

Cancer has developed multiple mechanisms to escape regulated growth and avoid apoptosis. Thus, the use of whole cell extracts, which contain several components having different possible intracellular targets, may provide an advantage over using one isolated plant compound. As most of the plants tested have a long history of oral use, particularly* Urtica* and* Origanum* species, and appear to be nontoxic, the potential of developing whole plant extracts for cancer treatment alone, in combination with other medications or as a possibly preventative treatment by addition to daily diet is promising. Nevertheless before any specific human use for cancer these plants should be further tested including additional* in vivo* studies in animal models and in human clinical trials.

## 6. Conclusions

Whole cell extracts (ethanol extraction) from* Urtica membranacea* (Urticaceae),* Artemesia monosperma* (Asteraceae), and* Origanum dayi post* (Labiatae), plants indigenous to the coastal plain and desert areas of Israel, exhibited dose- and time-dependent killing capabilities on various human derived hematological and solid tumor cell lines and primary cultures established from patients' biopsies. The killing activity was specific toward tumor cells, as the plant extracts had no effect on primary cultures of healthy human cells. Cell death caused by the whole plant extracts was via apoptosis. Plant extract from* Urtica membranacea* showed particularly strong anticancer capabilities since it inhibited actual tumor progression in a breast adenocarcinoma mouse model. Our results suggest that whole plant extracts are promising anticancer reagents.

## Supplementary Material

Supplementary Material describes the screen of 17 plants that were initially selected and tested for anti-cancer activity based on their historical and traditional use in treating cancer. This screen was the basis for selecting the most effective plant extracts that were further studied in details for their anti-cancer activity.

## Figures and Tables

**Table 1:** Human tumor cells used in the study.

Cell line	Type
LNCaP	Prostate adenocarcinoma
Colo205	Colon carcinoma
Hec-1A	Endometrial adenocarcinoma
OVCAR 3	Ovarian carcinoma
HepG2	Hepatocellular carcinoma
MCF-7	Breast ductal carcinoma
293	Embryonic kidney adenocarcinoma
Karpas 299	T cell, non-Hodgkin's lymphoma
A549	Alveolar basal epithelial adenocarcinoma-lung
SU-DHL-1	Anaplastic large cell lymphoma
YC	Normal EBV transformed lymphoblasts
OSTRA	Normal EBV transformed lymphoblasts
HUT 102	T cell lymphoma
T24P	Urinary bladder carcinoma
