# Supplementary material for: Evaluating Medicinal Plants for Anticancer Activity
Source: ScientificWorldJournal. 2014 Nov 13;2014:721402. doi: 10.1155/2014/721402 (PMC4248331; doi:10.1155/2014/721402)
Supplement: Supplementary file 1 — Supplementary Material describes the screen of 17 plants that were initially selected and tested for anti-cancer activity based on their historical and traditional use in treating cancer. This screen was the basis for selecting the most effective plant extracts that were further studied in details for their anti-cancer activity. [file 721402.f1.pdf]

**Figure S1: Effect of various whole plants extracts on growth of human tumor and healthy control cell lines**

**A-C:** Whole plant extracts (ethanol extracted) were prepared, coded as plant extracts no's 1-17 and tested for their potential as anti-cancer reagents. Various human tumor or control, healthy cell lines (Table 1; $10^5$ cells/100 μl) were grown with 3 mg/ml (final concentration) of the various whole plant extracts for 72 h, after which cell viability was determined as described in Materials and Methods. Results are expressed as percentage of death caused by each plant extract as compared to control cells (treated with DMSO/PBS at the same concentration as the plant extracts). Each column represents one representative out of 2–4 independent experiments performed.

**Figure S1**

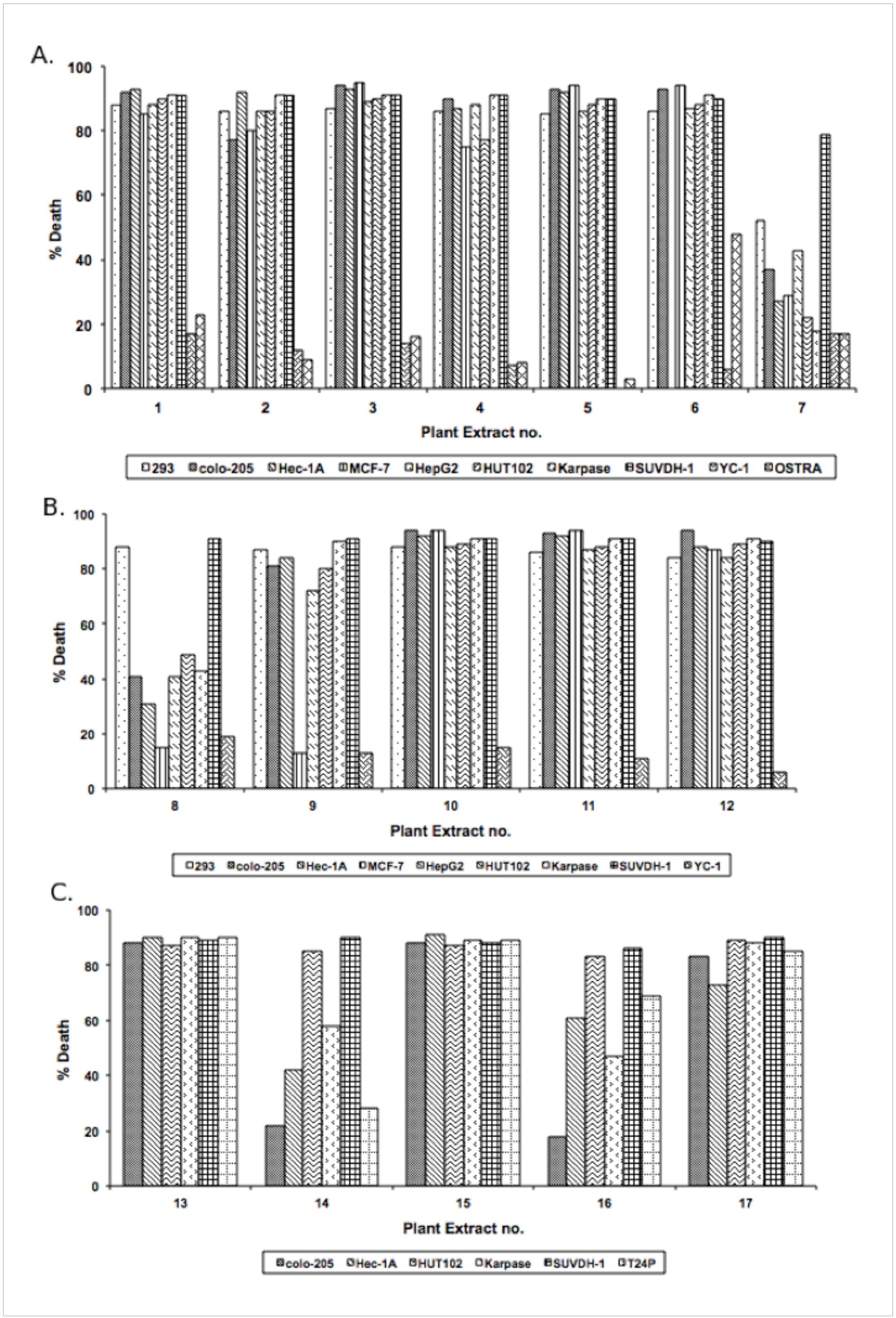
A.
% Death
0
20
40
60
80
100
1
2
3
4
5
6
7
Plant Extract no.
293 colo-205 Hec-1A MCF-7 HepG2 HUT102 Karpase SUVDH-1 YC-1 OSTRA
B.
% Death
0
20
40
60
80
100
8
9
10
11
12
Plant Extract no.
293 colo-205 Hec-1A MCF-7 HepG2 HUT102 Karpase SUVDH-1 YC-1
C.
% Death
0
20
40
60
80
100
13
14
15
16
17
Plant Extract no.
colo-205 Hec-1A HUT102 Karpase SUVDH-1 T24P
